# A PI3K-calcium-Nox axis primes leukocyte Nrf2 to boost immune resilience and limit collateral damage

**DOI:** 10.1083/jcb.202203062

**Published:** 2023-03-30

**Authors:** Giuliana D. Clemente, Helen Weavers

**Affiliations:** 1 School of Biochemistry, Biomedical Sciences, University of Bristol, Bristol, UK

## Abstract

Phagosomal reactive oxygen species (ROS) are strategically employed by leukocytes to kill internalized pathogens and degrade cellular debris. Nevertheless, uncontrolled oxidant bursts could cause serious collateral damage to phagocytes or other host tissues, potentially accelerating aging and compromising host viability. Immune cells must, therefore, activate robust self-protective programs to mitigate these undesired effects, and yet allow crucial cellular redox signaling. Here, we dissect in vivo the molecular nature of these self-protective pathways, their precise mode of activation, and physiological effects. We reveal *Drosophila* embryonic macrophages activate the redox-sensitive transcription factor Nrf2 upon corpse engulfment during immune surveillance, downstream of calcium- and PI3K-dependent ROS release by phagosomal Nox. By transcriptionally activating the antioxidant response, Nrf2 not only curbs oxidative damage but preserves vital immune functions (including inflammatory migration) and delays the acquisition of senescence-like features. Strikingly, macrophage Nrf2 also acts non-autonomously to limit ROS-induced collateral damage to surrounding tissues. Cytoprotective strategies may thus offer powerful therapeutic opportunities for alleviating inflammatory or age-related diseases.

## Teaser

Macrophages activate the cytoprotective factor Nrf2 upon phagocytic ROS production via PI3K-calcium-Nox signaling during immune surveillance. This protective response is crucial to sustain vital immune functions and limit macrophage acquisition of senescence-like features. Macrophage Nrf2 also acts non-autonomously to limit bystander damage to adjacent healthy tissues.

## Graphical Abstract

**Figure:**
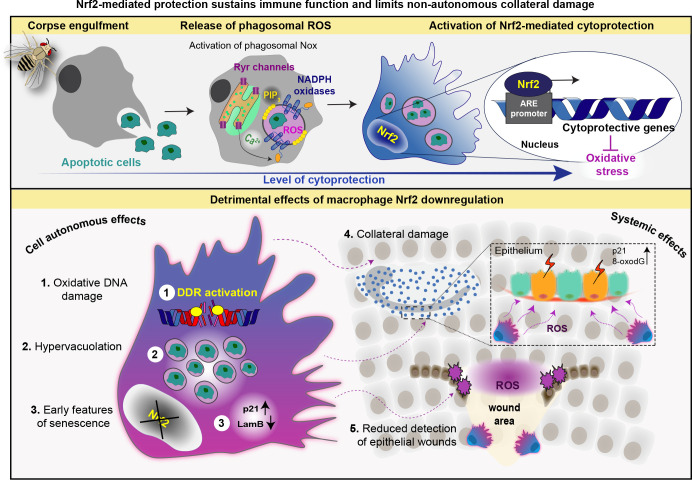

## Introduction

Tissues are constantly exposed to highly reactive and potentially toxic metabolites, such as reactive oxygen species (ROS) derived from cellular metabolism and physiology, or generated upon exposure to radiation or noxious chemicals (Finkel and Holbrook, 2000). Given the uncontrolled accumulation of cellular damage (including oxidative damage to cellular biomolecules) is considered to be a leading cause of tissue damage and aging (Gladyshev, 2014; Liochev, 2013), cells must employ powerful cytoprotective strategies to mitigate indiscriminate, deleterious effects of ROS (and other toxic byproducts) and shield tissues from potential injury (Bise et al., 2019; Burbridge et al., 2021; Telorack et al., 2016; Weavers et al., 2019). Cytoprotective strategies may thus be crucial in vivo to sustain tissue health and delay a progressive decline in tissue function.

Whilst mitochondria-derived ROS have received much attention in relation to tissue damage and aging (Balaban et al., 2005), in fact, NADPH and Dual oxidases (NOX and DUOX, respectively) represent the major source of endogenous ROS (Lambeth, 2004). Dual oxidases are expressed in several cell types including the epithelium, where moderate ROS release following injury steers immune cells toward sites of damage (Niethammer, 2016; Razzell et al., 2013; Yoo et al., 2011) and modulates enzymatic activities involved in healing (Hunter et al., 2018). Nevertheless, uncontrolled oxidative damage could hinder repair, thus damaged tissues upregulate cytoprotective pathways to minimize collateral damage and ensure effective healing (Telorack et al., 2016; Weavers et al., 2019).

NOX enzymes have been best characterized in cells of the innate immune system. Phagocytes voraciously engulf unwanted particles (e.g., dysfunctional “self” or dangerous intruders) and degrade them through a rapid NOX-dependent “burst” of ROS (Weavers and Martin, 2020). This local, sharp increase in ROS is not only an important antimicrobial weapon but can also activate phagosomal proteases to enhance proteolytic digestion (Reeves et al., 2002). Paradoxically, given their highly reactive and indiscriminate nature, ROS could cause substantial collateral damage to both the phagocyte and surrounding tissues. Nevertheless, leukocytes exhibit an unprecedented “resilience” to this hostile environment, suggesting they must be empowered with robust ROS detoxification strategies to preserve immune function, and yet retain sensitivity to low ROS levels for signaling. However, the exact identity of these protective strategies, their molecular regulation, and physiological significance remain largely unclear.

Comprehensive profiling of immune cytoprotective responses will help inform the development of novel strategies to enhance immunological vigor (i.e., the long-term maintenance of immune function; Hirokawa Katsuiku, 2019). Indeed, the ability to effectively buffer ROS is known to decline with age (Meng et al., 2017), suggesting that the activation of protective responses might become compromised. Repeated cycles of oxidative injuries could reduce the effectiveness of cytoprotective mechanisms and eventually trigger immune senescence, which in itself greatly increases the risk of degenerative disorders, neoplasia, and chronic infections (Akbar and Gilroy, 2020; Nikolich-Žugich, 2018; Shaw et al., 2013).

*Drosophila* has recently emerged as an invaluable in vivo system to explore the pathway(s) conferring “resilience” to oxidative stress, particularly in vulnerable epithelial tissues following injury (Burbridge et al., 2021; Mundorf et al., 2019; Weavers et al., 2019). Here, we characterize the cytoprotective mechanism(s) that increase *Drosophila* macrophage tolerance to oxidative stress during immune surveillance and inflammatory migration. We find that macrophages activate Nrf2 downstream of a conserved calcium-PI3K-Nox signaling axis to minimize the deleterious effects of phagosomal ROS. Strikingly, the activation of macrophage Nrf2 not only autonomously sustains multiple immune functions and delays the acquisition of cellular features typically associated with early senescence, but also plays a crucial role in minimizing ROS-associated bystander damage to surrounding (otherwise healthy) tissues. Further characterization of the cytoprotective strategies that boost innate immunity will no doubt open novel routes to intervene in immune decline and alleviate the morbidities of age-related disease.

## Results and discussion

### Macrophages activate Nrf2 downstream of apoptotic corpse uptake

**Figure 1.**
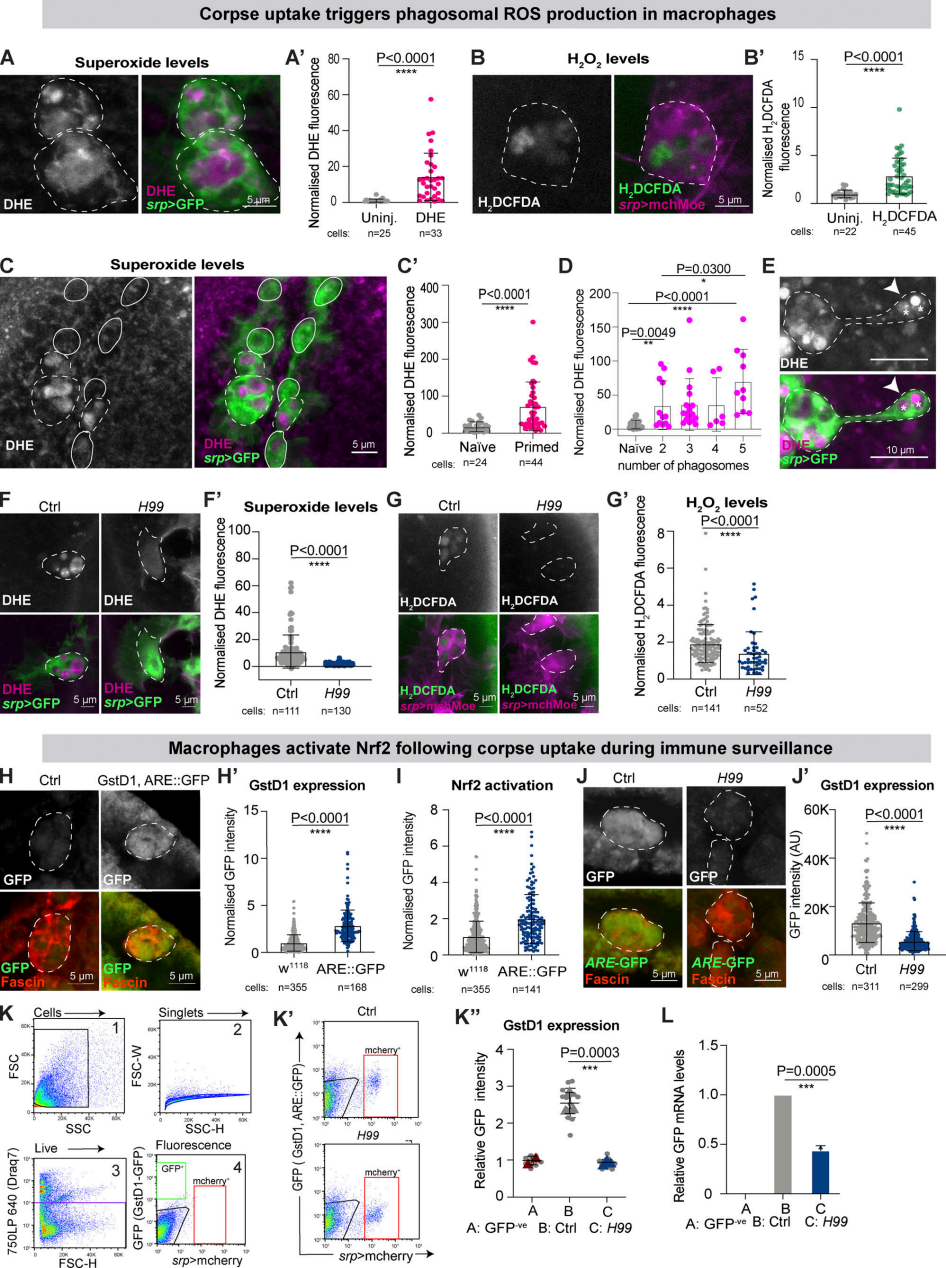
**Corpse uptake drives an oxidative burst and downstream Nrf2 activation in *Drosophila* macrophages. (A and B)** ROS production in stage 15 macrophage, revealed by DHE (magenta, A) and H_2_DCFDA (green, B). **(C and D)** ROS levels (DHE) increased upon apoptotic corpse uptake. In C, solid and dashed outlines indicate naïve and primed macrophages in stage 12 embryos, respectively. In D, levels of superoxide at the macrophage cell body are plotted against the number of cytoplasmic vacuoles. **(E)** ROS release (white arrow) detected at nascent phagosomes (white asterisks) in stage 13 embryos. **(F and G)** Decreased ROS levels in stage 15 *H99* macrophages, as detected by DHE (F) and H_2_DCFDA (G). **(H and I)** Stage 15 embryonic macrophages activated *ARE*-GFP reporters of Nrf2 activation (green) including the GstD1, ARE-GFP reporter (H′), or a synthetic ARE-GFP reporter for Nrf2 activity (I). **(J)** Decreased Nrf2 activation in stage 15 *H99* macrophages using GstD1, ARE-GFP reporter. **(K)** FACS plots showing the gating strategy adopted on cell suspension from non-transgenic *w*^*1118*^ *Drosophila* embryos to eliminate background fluorescence. Stage 15 *H99* macrophages showed reduced activation of the GstD1, *ARE*-GFP reporter. **(L)** Relative GFP mRNA levels from sorted stage 15 macrophages of the indicated genotypes; data from two independent biological replicates. In all figures, the cell body is indicated by a dashed or solid white outline. Macrophages labeled in red (fascin, H, J; *srp* > mchMoesin, B, G) or green (*srp* > GFP, A, C, E, and F). ROS detected with DHE (magenta, A, C, E, and F) or H_2_DCFDA (green, G). ns: not significant, **P < 0.01, ***P < 0.001, ****P < 0.0001 via Mann–Whitney test (A′, A″, B′, C′, E′, F′, G′, H′, I, J′) or one-way ANOVA followed by Dunnett’s multiple comparison analysis (K″, L). Images collected from 7 uninjected and 5 injected embryos (A); 3 uninjected and 7 embryos (B); 8 embryos (C); 14 control and 18 *H99* embryos (F); 26 control and 10 *H99* embryos (G); 17 control and 10 GstD1, ARE-GFP embryos (H); 12 control and 10 *H99* embryos (J).

**Figure S1.**
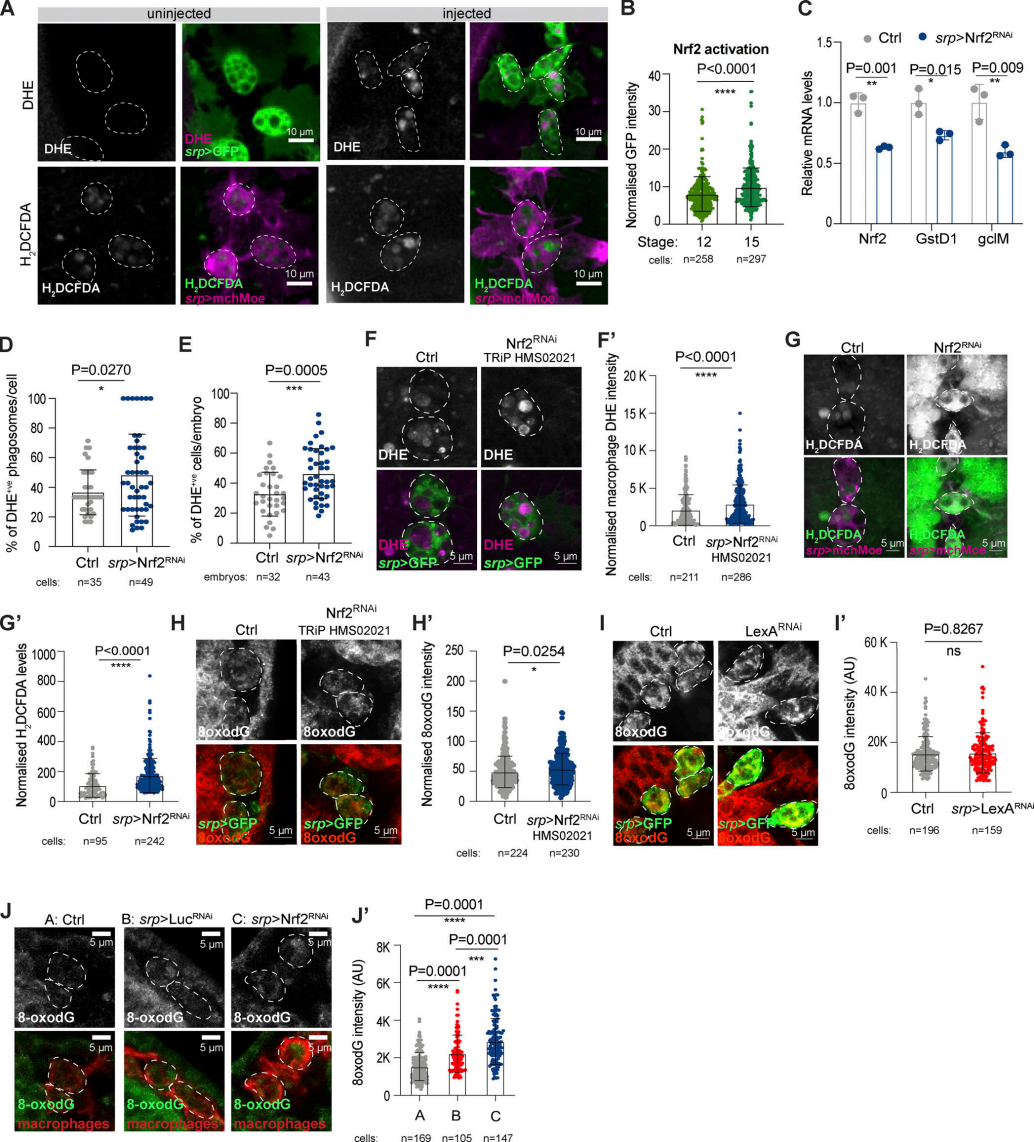
**Activation of Nrf2 protects embryonic macrophages from excessive ROS accumulation. (A)** Representative images of macrophages from uninjected embryos or embryos injected with fluorescent ROS dyes. **(B)** Macrophage *ARE*-GFP activation increased with embryonic stage, using a synthetic *ARE*-GFP reporter for Nrf2 activity. **(C)** Relative *Nrf2*,* GstD1*, and *gclM* mRNA levels from stage 15 control and *srp* > Nrf2^RNAi^ macrophages isolated by FACS; data representative of three technical replicates of one biological replicate. **(D and E)** Stage 15 macrophage Nrf2^RNAi^ increased the percentage of DHE-positive phagosomes per cell (D) and DHE-positive macrophage per embryo (E). **(F–H)** Nrf2^RNAi^ increased the levels of ROS at the cell body (F and G) and increased oxidative damage (H). **(I)** Macrophage expression of bacterial LexA^RNAi^ did not increase DNA oxidative damage (8-oxodG) above control levels. **(J)** Macrophage expression of Nrf2^RNAi^ increases DNA oxidative damage (8-oxodG) above Luciferase^RNAi^ and control levels. Data (C–E, G, and J) generated using Nrf2^RNAi^ Flybase ID FBtp0069370 and data (F and H) generated using Nrf2^RNAi^ TRiP HMS02021. *P < 0.05, **P < 0.01, ***P < 0.001, ****P < 0.0001 via Mann–Whitney test (B, F′, G′, H′, I′) and unpaired *t* test (C, D, and E), Dunn’s multiple comparison (J′). Images collected from 17 control and 8 *ARE*-GFP embryos (B); 8 control, 9 *srp* > Nrf2^RNAi^ embryos (D); 21 control, 30 *srp* > Nrf2^RNAi^ embryos (F); 10 control, 24 *srp* > Nrf2^RNAi^ embryos (G); 7 control, 8 *srp* > Nrf2^RNAi^ embryos (H); 5 control, 5 *srp* > LexA^RNAi^ embryos (I); 5 control, 5 Luciferase^RNAi^, 7 *srp* > Nrf2^RNAi^ embryos (J). Also see Fig. 1.

Professional phagocytes, such as neutrophils and macrophages, release ROS during the “respiratory burst” as a potent microbiocidal weapon (Fang, 2011; Herb and Schramm, 2021; Slauch, 2011). Here, microinjection of *Drosophila* embryos with fluorescent dyes for superoxide (DHE) and hydrogen peroxide (H_2_DCFDA; Soh, 2006) revealed ROS production at the phagosomes of stage 15 embryonic macrophages, even in the absence of bacterial infection or laser-induced tissue damage (Fig. 1, A and B; and Fig. S1 A).

Professional phagocytes not only orchestrate the removal of invading pathogens but also of dying cells (Weavers and Martin, 2020). Therefore, the production of phagosomal ROS (Fig. 1, A and B) might correlate with the intense apoptotic corpse engulfment by macrophages in the early stages of *Drosophila* embryogenesis (Weavers et al., 2016a). In fact, phagocytes facilitate crucial tissue sculpting by engulfing dying cells from surrounding tissues, a macrophage function well-conserved from flies to mammals (Jacobson et al., 1997; Kerr et al., 1972; Wood et al., 2000). Intriguingly, this corpse clearance “primes” the macrophages for future, robust inflammatory behavior (Weavers et al., 2016a). At embryonic stage 12, a subset of macrophages had already cleared apoptotic corpses as indicated by the presence of cytosolic vacuoles (Fig. 1 C, dashed outlines). Within the same embryo, many macrophages were still “naïve” and yet to phagocytose any corpses (Fig. 1 C, solid outlines). Strikingly, “primed” macrophages strongly accumulated superoxide, proportionally to the number of phagosomes within the cell body, suggesting that corpse engulfment drove an oxidative burst (Fig. 1, C′ and D). Macrophages extend long cytoplasmic protrusions (“pseudopods”) for the long-range uptake of cellular debris (Weavers et al., 2016a). We observed the release of ROS on nascent phagosomes at the very tip of pseudopods (Fig. 1 E), reinforcing the correlation between corpse uptake and the oxidative burst. We also analyzed embryos homozygous for the chromosomal deletion *Df(3L)H99,* which removes the proapoptotic genes *hid*, *grim*, and *reaper*, resulting in embryos completely lacking apoptosis (White et al., 1996); *H99* macrophages were “naïve,” having not phagocytosed apoptotic corpses (Weavers et al., 2016a) and exhibited a marked decrease in ROS production (Fig. 1, F and G).

Given that uncontrolled and prolonged ROS exposure could lead to the progressive accumulation of oxidative damage (Pizzino et al., 2017), we speculated that macrophages could employ cytoprotective pathways to control phagosomal ROS during immune surveillance, even in the absence of infection. The cap-and-collar (CNC) transcription factor nuclear factor erythroid derived-2 (Nrf2) controls the expression of genes involved in redox balance by binding to their antioxidant responsive elements (AREs; Schäfer and Werner, 2015). In *Drosophila*, two independent transcriptional reporters are available to monitor Nrf2 activation; one reporter expresses GFP under the control of synthetic *ARE* repeats, providing a general readout of Nrf2 activation (Chatterjee and Bohmann, 2012), whilst a second reporter expresses GFP under the control of the GstD1 promoter, a well-known downstream target of Nrf2 (Sykiotis and Bohmann, 2008). *Drosophila* embryonic macrophages strongly activated both reporters of Nrf2 *ARE* binding at mid-embryogenesis, suggesting robust activation of the Nrf2-regulated antioxidant response (Fig. 1, H and I).

Next, we tested whether the Nrf2 signaling pathway was activated in macrophages downstream of corpse uptake. For this, we measured activation of the GstD1, *ARE*-GFP reporter in *H99* mutant macrophages; these naïve macrophages not only exhibited reduced phagosomal ROS production (Fig. 1, F and G) but also significantly reduced activation of Nrf2 compared to controls, as shown by immunofluorescence (Fig. 1, J and J’), flow cytometry (Fig. 1, K and K″), and RT-qPCR (Fig. 1 L). These data suggest that the phagocytosis-dependent release of ROS is a key trigger of the Nrf2-mediated antioxidant response in macrophages during embryonic immune surveillance in vivo. The Nrf2 response has been recently detected within murine macrophages following bacterial infection (Wang et al., 2019). Nevertheless, the mechanisms controlling Nrf2 activation independently of pathogen encounter and its role in preserving vital immune activities remains largely unclear.

### Nrf2 induces resilience to oxidative stress in *Drosophila* macrophages

**Figure 2.**
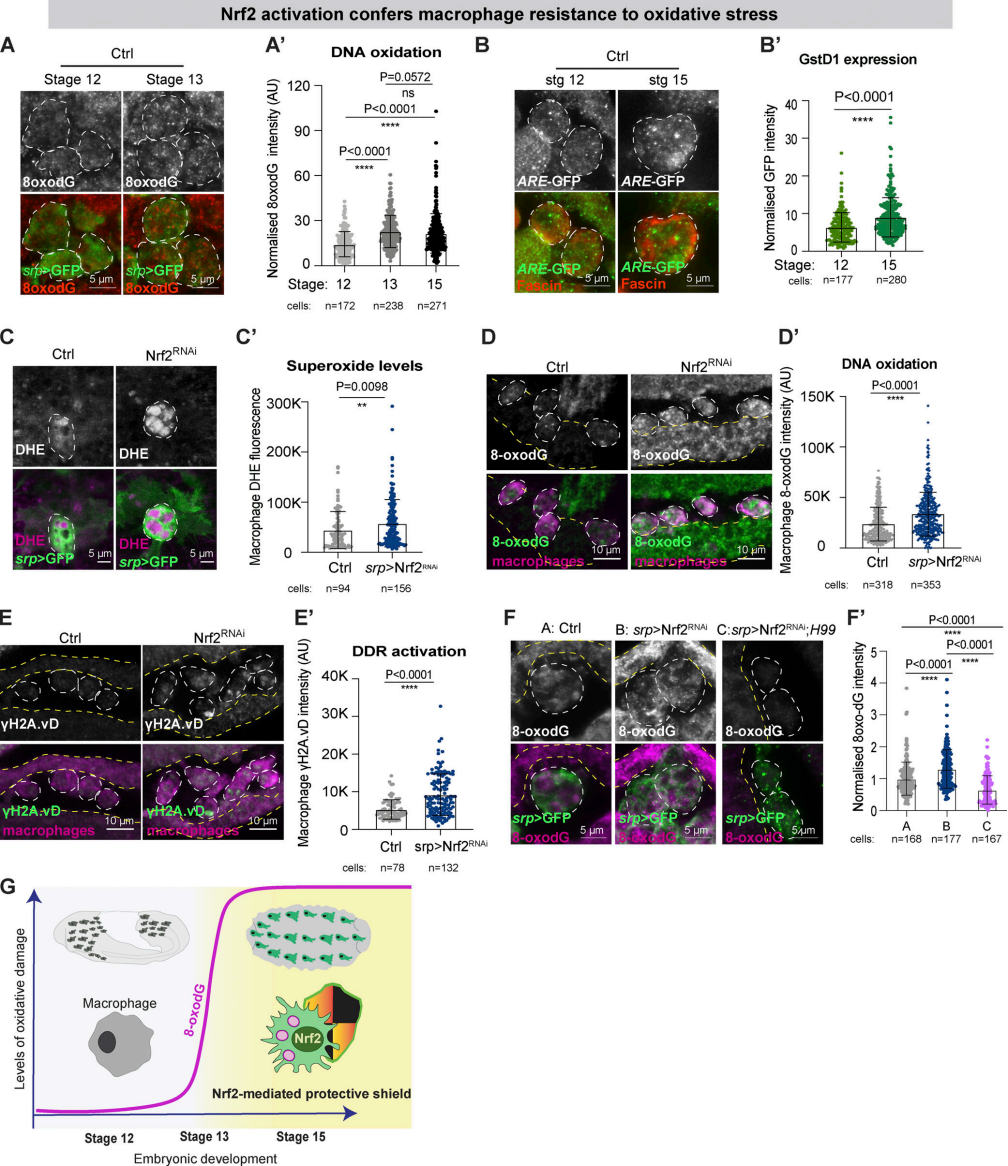
**Macrophage Nrf2 confers resistance to oxidative stress. (A and B)** DNA oxidation (8oxodG, red, A) and *ARE*-GFP activation (green, GstD1, *ARE*-GFP reporter, B) increased as embryonic macrophages matured. **(C)** ROS levels (DHE, magenta) increased in stage 15 *srp* > Nrf2^RNAi^ macrophages. **(D and E)** stage 15 *srp* > Nrf2^RNAi^ macrophages accumulated oxidative damage (8oxodG, D and D′) and DNA damage (H2AvD, E and E′). **(F)** The *H99* mutation rescued the accumulation of DNA oxidative damage (8-oxodG) caused by corpse uptake and exacerbated by down-regulation of Nrf2. **(G)** Schematic of Nrf2 activation within macrophages. In all figures, the cell body is indicated by a dashed white outline. Macrophages labeled in green (*srp* > GFP, A, C, and F) or red (anti-fascin, B and E) *srp* > GFP, D). Adjacent epithelium is marked by yellow dashed lines (D, E, and F). Data (C, D, E, and F) were generated using Nrf2^RNAi^ Flybase ID FBtp0069370. ns: not significant, **P < 0.01, ****P < 0.0001 via Mann–Whitney test (A′, B′, C′, D′, E′) or one-way ANOVA followed by Krushal–Wallis comparison analysis (F′). Images were collected from: 9 stage 12, 10 stage 13, 10 stage 15 embryos (A); 8 stage 12, 10 stage 15 embryos (B); 11 control and 17 *srp* > Nrf2^RNAi^ embryos (C); 28 control and 29 *srp* > Nrf2^RNAi^ embryos (D); 9 control and 10 *srp* > Nrf2^RNAi^ embryos (E); 5 control, 5 Nrf2^RNAi^ and 7 Nrf2^RNAi^; *H99* embryos (F).

We next sought to test whether Nrf2 fine-tunes macrophage ROS levels to minimize potentially debilitating oxidative damage. Given that the release of phagosomal ROS was dependent on corpse uptake during developmental dispersal (Fig. 1, F and G), we examined whether wild-type macrophages accumulated progressively higher levels of oxidative damage as they transitioned through embryonic development. We assessed oxidative damage in the form of oxidized guanine (8-oxodG, Fig. 2 A), a known biomarker of mutagenesis caused by oxidative stress (Ock et al., 2012). Intriguingly, accumulation of oxidative damage correlated with macrophage maturation even in wild-type embryos (Fig. 2, A and A′). This stage-dependent increase in macrophage oxidation was paralleled by marked activation of Nrf2 by stage 15 (Fig. 2, B and B′; and Fig. S1 B). Intriguingly, oxidative damage did not increase significantly from stage 13 to 15 (Fig. 2 A′), which perhaps reflects the robust Nrf2 activation at this time.

To further explore the role of Nrf2 in buffering intracellular ROS, we inhibited macrophage Nrf2 using multiple independent RNAi lines and the macrophage-specific driver *serpent*-Gal4. Genetic inhibition of Nrf2 attenuated the macrophage antioxidant response, as shown by reduced expression of *GstD1* and *gclM*, key Nrf2 targets (Fig. S1 C). Macrophage Nrf2-RNAi (Fig. S1 C) caused significantly increased ROS levels within the cell body as well as a marked increase in the total number of DHE-positive phagosomes (superoxide, Fig. 2 C and Fig. S1, D–F; hydrogen peroxide, Fig. S1, G and G′). Nrf2^RNAi^ macrophages also strongly accumulated oxidative damage (Fig. 2, D and D′; and Fig. S1, H and H′; Ock et al., 2012) and activated the DNA damage response (DDR) as shown by increased phospho-H2AvD (the *Drosophila* phospho-H2AX equivalent, Fig. 2, E and E′). The dramatic accumulation of damage was specific to Nrf2 downregulation, as Nrf2^RNAi^ caused a much stronger accumulation of 8-oxodG adducts within macrophages compared with the expression of unrelated RNAi constructs (LexA^RNAi^, Fig. S1 I, and Luciferase^RNAi^, Fig. S1 J).

To explore whether the elevated oxidative damage in Nrf2^RNAi^ macrophages was directly caused by phagosomal ROS, we analyzed DNA oxidation in Nrf2^RNAi^ macrophages from *H99* mutant embryos. Here, genetic inhibition of corpse uptake (via *H99*) rescued the accumulation of oxidative damage caused by macrophage Nrf2^RNAi^ (Fig. 2, F and F′). These data suggest that Nrf2-dependent resilience counteracts the harmful consequences of ROS accumulation downstream of corpse uptake in *Drosophila* macrophages (Fig. 2 G). Interestingly, monocytes isolated from blood donors are more sensitive to oxidative DNA lesions than their more mature macrophage descendants (Ponath and Kaina, 2017); this differential vulnerability to ROS is consistent with our findings that redox-stress responses are activated upon differentiation to maximize self-protection and boost the fitness of these long-lived cells.

### Macrophages activate Nrf2 downstream of calcium and PI3K-dependent Nox activity

**Figure 3.**
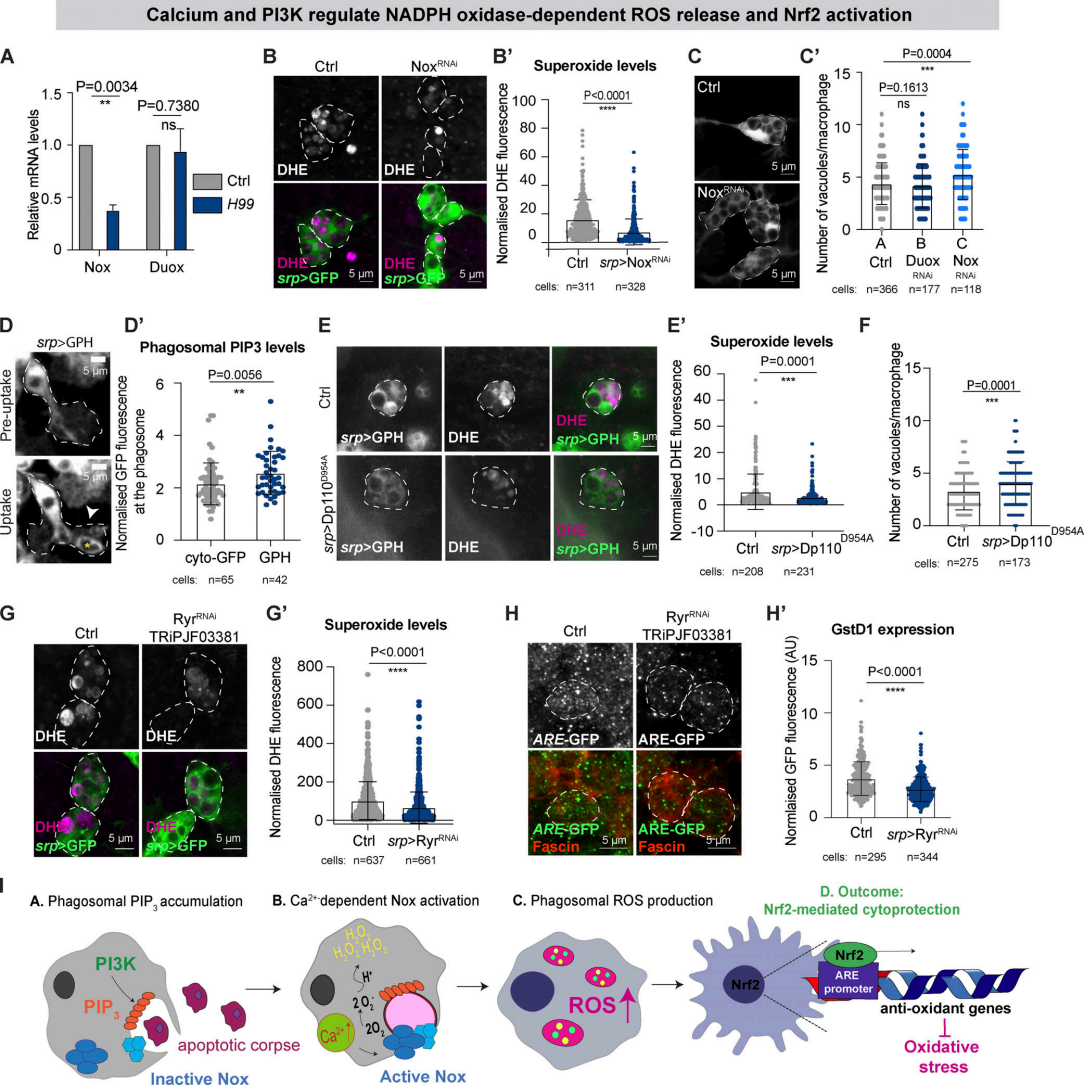
**Nox-dependent release of ROS is regulated by PI3K and calcium. (A)** RT-qPCR data from sorted stage 15 control and *H99* macrophages (two independent biological replicates). **(B and C)** *srp* > Nox^RNAi^ decreased phagosomal ROS (B) and caused hypervacuolation (C). **(D and D′)** Macrophage PIP_3_ (*srp* > GPH, white arrowhead) accumulated on nascent phagosomes (yellow asterisk) during embryonic stage 13. The image is a still from a movie of macrophage dispersal during stage 13 of development (see Video 1). **(E and E′)** Phagosomal ROS levels (DHE, magenta) in *srp* > Dp110^D954A^ macrophages. **(F)** Macrophage-specific inhibition of PI3K (*srp* > Dp110^D954A^) caused hypervacuolation. **(G and H)** Inhibition of calcium release by *srp* > Ryr^RNAi^ reduced phagosomal ROS (G and G′ DHE, magenta) and Nrf2 activation (H and H′, GstD1, *ARE*-GFP reporter). **(I)** Schematic showing corpse uptake promotes PIP_3_ and calcium-dependent Nox activation, driving ROS release and activation of Nrf2-mediated macrophage cytoprotection. In all figures, with the exception of D, images are representative of stage 15 macrophages. In all figures, the cell body is indicated by a white dashed outline. Macrophages labeled in red (anti-fascin, H) or green (B, C, G *srp* > GFP; D, E *srp* > GPH). ns: not significant, **P < 0.01, ***P < 0.001, ****P < 0.0001 via Mann–Whitney test (B′, C, D′, E′, F, G′, H′) or unpaired *t* test (A). Images collected from 26 control and 26 *srp* > Nox^RNAi^ embryos (B); 51 control, 33 *srp* > Duox^RNAi^, and 27 *srp* > Nox^RNAi^ embryos (C); 20 control and 35 srp > Dp110^D954A^ embryos (E); 57 control and 68 *srp* > Ryr^RNAi^ embryos (G); 7 control and 11 *srp* > Ryr^RNAi^ embryos (H).

**Figure S2.**
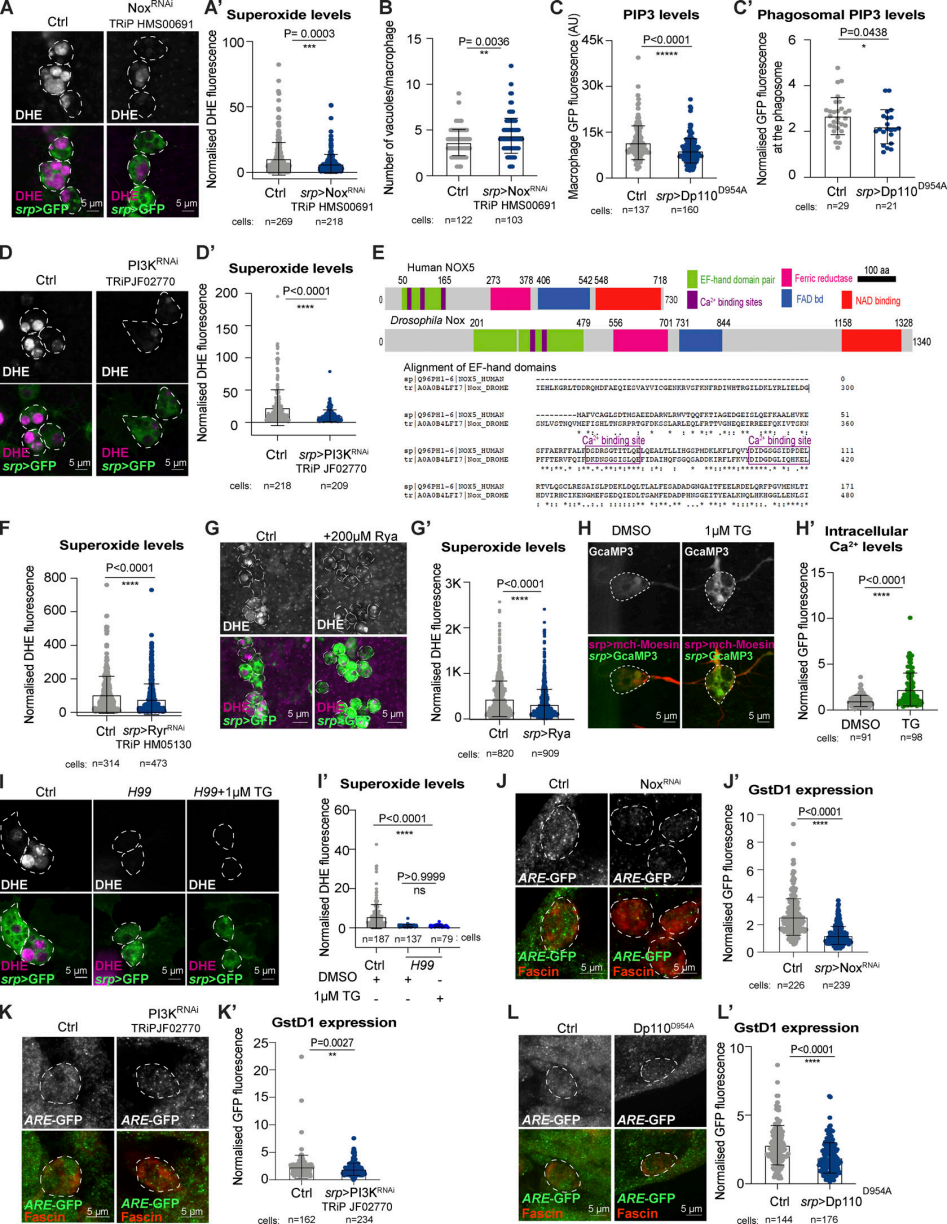
**A PI3K, Calcium, and NOX axis drive macrophage Nrf2 activation. (A and B)** Expression of Nox^RNAi^ decreased levels of ROS at the cell body in stage 15 macrophages (A and A′) and caused hypervacuolation (B). **(C)** Overexpression of Dp110^D954A^ reduced levels of total cellular and phagosomal-localized PIP3 in stage 13 macrophages. **(D)** Overexpression of PI3K^RNAi^ decreased levels of ROS at the cell body in stage 15 macrophages. **(E)** Domain organization of *Drosophila* Nox and human NOX5 as predicted using Interproscan (Blum et al., 2021). Both proteins have a highly conserved, calcium-sensitive EF-hand domain at the N-terminus as shown by sequence alignment. **(F)** *srp* > Ryr^RNAi^ reduced phagosomal ROS in stage 15 macrophages using RNAi construct TRiPHM05130. **(G)** Inhibitory concentrations of Ryanodine significantly decreased superoxide production within stage 13 macrophages. **(H)** Exposure of stage 15 embryos to 1 μM Thapsigargin (TG) induced the release of calcium in macrophages. **(I)** Thapsigargin did not rescue phagosomal ROS production by stage 15 *H99* macrophages. **(J–L)** Reduced dNrf2 activation was observed upon Nox^RNAi^ (J and J′), PI3K^RNAi^ (K and K′) and overexpression of Dp110^D954A^ (L and L′) in stage 15 macrophages. Cell bodies are indicated by dashed outline. Macrophages labeled in green (*srp* > GFP, A, D, G, and I) or red (srp > mchMoesin, H; fascin, J, K, and L). ROS production detected with DHE (in magenta, A, D, G, and I). dNrf2 activation evaluated using GstD1, ARE-GFP reporter (green, J, K, and L) *P < 0.05, ****P < 0.0001 via Mann–Whitney test (A′, B, C, D′, F, G′, H′, J′, K′, and L′). Images collected from: 26 control and 22 *srp* > Nox^RNAi^ embryos; 24 control and 24 *srp* > PI3K^RNAi^ embryos; 60 control and 57 *srp* > Ryr^RNAi^ embryos (F); 98 control and 51 Ryanodine-treated embryos (G); 12 DMSO only and 10 *1*μM TG embryos (H); 24 control and 19 *H99* and 10 TG-treated *H99* embryos (I); 10 control and 10 *srp* > Nox^RNAi^ embryos (J); 7 control and 7 *srp* > PI3K^RNAi^ embryos (K); 5 control and 7 *srp* > Dp110^D954A^ embryos (L). Also see Fig. 3.

We next explored the molecular mechanisms underlying macrophage ROS release and the downstream activation of Nrf2. NADPH oxidases are well-conserved enzymes dedicated to the production of superoxide (Taylor and Tse, 2021). Although both classes of NADPH oxidases, Nox and Duox, were expressed within *Drosophila* macrophages, corpse uptake transcriptionally upregulated *Nox* expression, leaving levels of *Duox* relatively unchanged (Fig. 3 A). Moreover, downregulation of macrophage Nox, using multiple independent RNAi lines, drastically reduced the release of phagosomal superoxide (Fig. 3, B and B′; and Fig. S2, A and A′). Interestingly, Nox^RNAi^ (but not Duox^RNAi^) macrophages accumulated vacuoles within the cell body (Fig. 3 C and Fig. S2 B), suggesting that Nox-mediated ROS production could be key for the efficient processing of macrophage phagosomal content.

Phagosomal NADPH oxidases are tightly controlled to protect resting cells from dangerous exposure to unwanted ROS (Nathan and Cunningham-Bussel, 2013). A well-known marker of phagosome maturation is the progressive change in phospholipid composition (Swanson, 2014). Using a GFP-Pleckstrin homology (GPH) reporter, we monitored the accumulation of phosphoinositides PtdIns (3,4,5)P3 (PIP_3_) on the membrane of newly-formed phagosomes (Fig. 3, D and D′ and Video 1). Macrophage-specific expression of the mutated (catalytically inactive) phosphoinositide 3-kinase (PI3K) subunit Dp110^D954A^ (previously shown to act as an inhibitor of PI3K signaling [Wood et al., 2006]) not only reduced PIP_3_ accumulation at the cell body (*srp* > GPH, Fig. 3 E and Fig. S2 C) but also PIP_3_ enrichment at the phagosome (Fig. 3 E and Fig. S2 C′). Expression of Dp110^D954A^ or macrophage-specific RNAi of the PI3K catalytic subunit caused a dramatic decrease in the levels of superoxide (Fig. 3, E and E′; and Fig. S2, D and D′), suggesting that accumulation of PIP_3_ is required for the phagocytosis-dependent activation of Nox. Interestingly, inhibition of PI3K resulted in a hypervacuolation phenotype (Fig. 3 F), similar to that observed following Nox^RNAi^, again suggesting a role for ROS in the timely digestion of phagosomal content.

**Video 1.** **PIP3 accumulates on the membrane of a nascent phagosome.** Time-lapse imaging of *srp* > GPH macrophages migrating in a stage 13 embryo. A white asterisk marks the position of a macrophage that leaves the ventral midline to engulf an apoptotic corpse. Accumulation of PIP3 around the phagocytic cup is observed (white arrow). A maximum projection after a 3D Gaussian blur (FIJI, radius 0.8/0.8/1) of eight consecutive equatorial planes taken at 1 µm spacing is shown. Scale bar: 10 μm. Time stamp: minute. Related to Fig. 3.

**Figure S3.**
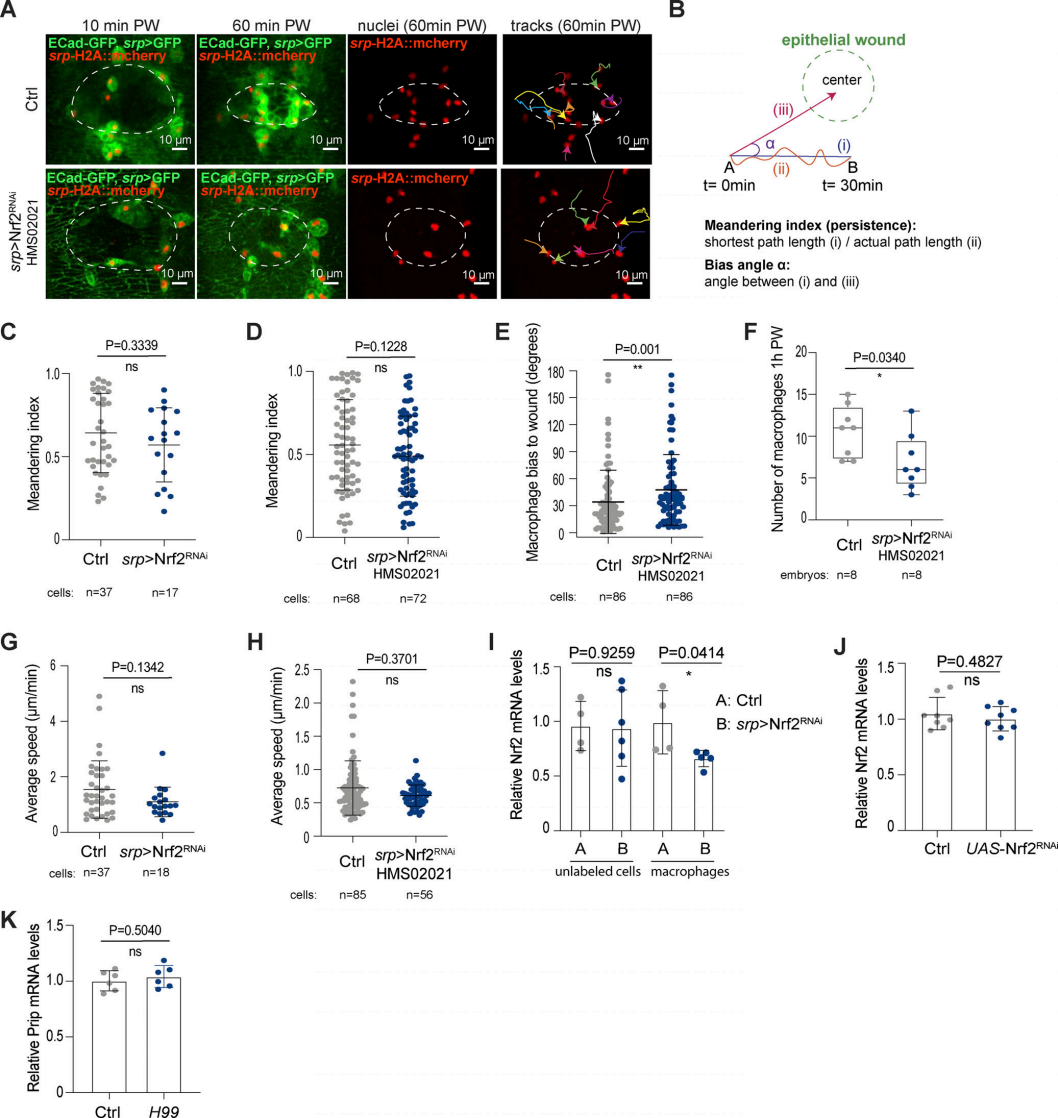
**Macrophage Nrf2 is required for the timely detection of epithelial wounds in vivo. (A)** Nrf2^RNAi^ macrophages exhibited reduced interest in sites of epithelial damage. **(B)** Definition of meandering index (persistence) and angle of migration (bias) towards epithelial wounds. **(C–H)** Quantification of meandering index (C and D), angle of migration (E), number of macrophages recruited to the site of damage 1-h post-wounding (F) and average speed (G and H) in control and *srp* > Nrf2^RNAi^ stage 15 macrophages. **(I)** Evaluation of *Nrf2* mRNA levels by RT-qPCR within embryonic macrophages and in cells other than macrophages in control and *srp > Nrf2-RNAi* embryos; data from two independent biological replicates. **(J)** Evaluation of *Nrf2* mRNA levels by RT-qPCR in control and UAS-Nrf2^RNAi^ embryos. **(K)** Evaluation of *Prip* mRNA levels by RT-qPCR in control and *H99* mutant embryos. In A, sites of epithelial damage are marked by a white dashed outline, macrophages are labeled in green (*srp* > GFP), macrophage nuclei are labeled in red (*srp*-H2A::mcherry). Data (C, G, I, and J) generated using Nrf2^RNAi^ Flybase ID FBtp0069370 and data (A, D–F, and H) generated using Nrf2^RNAi^ TRiP HMS02021. PW: post-wounding. ns: not significant, *, P < 0.05; **P < 0.01, ****P < 0.0001 via Mann-Whitney test (C, E, and H) or unpaired *t* test (D, F, and I). Data have been collected from 7 controls and 7 *srp* > Nrf2^RNAi^ embryos (C and G); 10 control and 9 *srp* > Nrf2^RNAi^ embryos (D and H); 9 control and 7 *srp* > Nrf2^RNAi^ embryos (E); 8 control and 8 *srp* > Nrf2^RNAi^ embryos (F). Also see Fig. 4.

*Drosophila* Nox is closely related to the mammalian calcium-sensitive enzyme NOX5 (HHPred [Söding et al., 2005] homology score e-value 1.9e^−80^ with human NOX5). Further, in silico analysis of *Drosophila* Nox revealed the presence of a N-terminal EF-hand calcium-binding domain (Fig. S2 E) that shared 88.7% similarities to the EF domain of the human ortholog (Sievers et al., 2011). In *Drosophila*, the uptake of apoptotic corpses triggers a rapid rise in intracellular calcium (Weavers et al., 2016a), likely mediated by release from intracellular stores via Ryanodine ER/SR Ca^2+^ channels (RyR receptors [Cuttell et al., 2008]). Given that *Drosophila* Nox possesses a conserved EF-hand domain, we tested for calcium-dependent regulation of Nox activity. Blocking the release of Ca^2+^ from the ER by macrophage-specific knockdown of RyR, or inhibitory doses of the drug Ryanodine, significantly decreased phagosomal ROS (Fig. 3, G and G′; and Fig. S2, F and G′), suggesting that Ca^2+^ positively regulated Nox activity. We also exposed embryos to 1 μM Thapsigargin to force Ca^2+^ release from the ER (Fig. S2, H and H′); however, the treatment of *H99* mutant embryos with Thapsigargin did not rescue the production of intracellular ROS (Fig. S3, I and I′), suggesting that Ca^2+^ was required but not sufficient to stimulate Nox activity.

Our data suggest that the activation of fully functional NADPH oxidases, such as *Drosophila* Nox, are under strict control, providing protection from potential exposure to unwanted ROS. However, there is a significant lack of knowledge on how NADPH oxidase activity modulates cytoprotection in vivo. Strikingly, multiple methods that dampened macrophage ROS levels (inhibition of Ca^2+^ release via Ryr^RNAi^, knockdown of Nox, or inhibition of Dp110) significantly decreased macrophage Nrf2 activation (Fig. 3 H and Fig. S2, J–L), suggesting that macrophages fine-tune their Nrf2-mediated antioxidant response according to the levels of phagosomal ROS production downstream of a conserved calcium-PI3K-Nox signaling axis (Fig. 3 I).

### Macrophage Nrf2 sustains proinflammatory behavior in vivo

**Figure 4.**
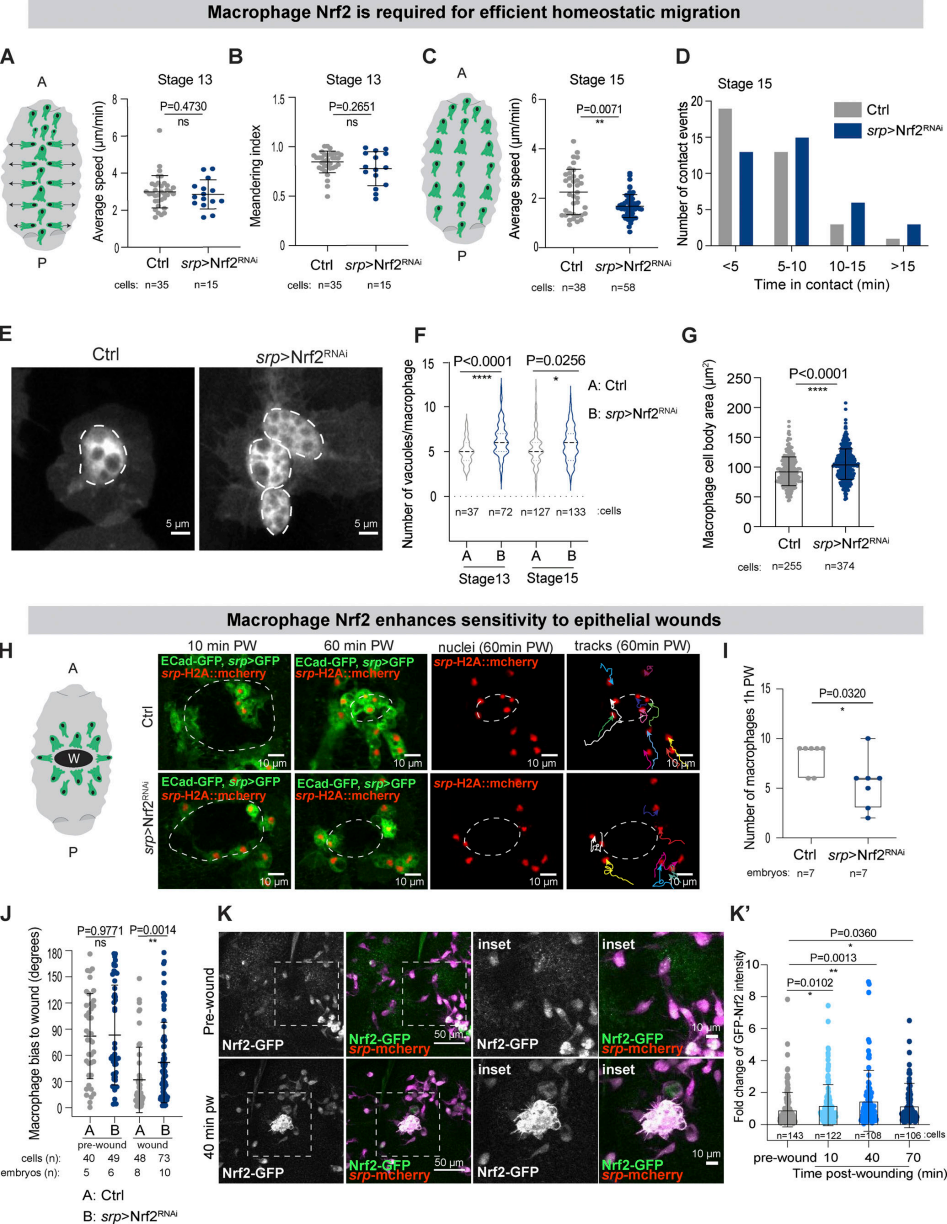
**Macrophage Nrf2 boosts homeostatic patrolling and pro-inflammatory behavior. (A and B)** Average speed and directionality of laterally migrating stage 13 control and *srp* > Nrf2^RNAi^ macrophages. **(C and D)** Average speed and CIL behavior of stage 15 control and *srp* > Nrf2^RNAi^ macrophages. **(E–G)** Nrf2 knockdown (*srp* > Nrf2^RNAi^) increased the number of phagosomes per macrophage and caused a modest increase in cell body size (hypertrophy). **(H and I)** Wound recruitment defect upon macrophage-specific Nrf2^RNAi^ analyzed at stage 15 of embryonic development. **(J)** Macrophage Nrf2^RNAi^ decreased the migrational bias toward sites of epithelial damage, as measured by the angle α relative to the center of the wound. **(K)** 18 h APF pupal macrophages strongly upregulated Nrf2 upon wounding. Cell bodies (E), site of epithelial wounds (H), and insets (K) are indicated by white dashed outlines. Macrophages labeled in green (*srp* > GFP, E, H) or magenta (*srp*-mcherry, K). Macrophage nuclei labeled in red (*srp* > H2A::mcherry, H), Nrf2 in green (Nrf2-GFP, K). All Nrf2^RNAi^ data were generated using Nrf2^RNAi^ Flybase ID FBtp0069370. PW: post-wounding. ns: not significant, *, P < 0.05; **P < 0.01, ****P < 0.0001 via Mann-Whitney test (A, B, C, F, G, J) or unpaired t-test (I), one-way ANOVA followed by Dunn’s comparison analysis (K). Images were collected from 5 control and 5 *srp* > Nrf2^RNAi^ embryos (A, B); 5 control and 5 *srp* > Nrf2^RNAi^ embryos (C); stage 13: 5 control and 12 *srp* > Nrf2^RNAi^ embryos and stage 15: 24 control and 33 *srp* > Nrf2^RNAi^ embryos (F); 44 control, 58 *srp* > Nrf2^RNAi^ embryos (G); 7 control and 7 *srp* > Nrf2^RNAi^ embryos (I, I′); 4 pupae (K).

Whilst our data suggest that macrophages activate Nrf2-mediated cytoprotection during immune surveillance to limit potentially deleterious oxidative damage, the importance of this protection for macrophage behavior in vivo remains unclear. Therefore, we investigated whether loss of Nrf2 affected the migratory behavior of leukocytes by live-imaging their dynamics in vivo. The developmental migration of *Drosophila* macrophages along the ventral nerve cord and their subsequent lateral movement away from the midline have been previously well characterized (Tepass et al., 1994; Wood et al., 2006). Inhibition of macrophage Nrf2 did not alter the speed and directionality of these early developmental migrations (Fig. 4, A and B), so by stage 15, the macrophages had reached their stereotypical positions (data not shown).

From stage 15, macrophage migration becomes more randomized and contact inhibition of locomotion (CIL) maintains their even distribution during immune surveillance (Davis et al., 2015). Macrophages lacking Nrf2 at this stage moved slower than controls (Fig. 4 C) and remained in contact for longer periods of time (Fig. 4 D), suggesting that they were less efficient in repolarizing their movement after contacting a neighboring cell. Mature stage 15 macrophages might rely more on Nrf2-mediated protection to sustain effective migration to counteract oxidative damage that has progressively accumulated during immune surveillance. Indeed, ROS are a known permissive signal for cell migration (Hurd et al., 2012), and through the oxidation of redox-sensitive enzymes, may act as a functional switch that allows fine control of migration. Robust ROS detoxification strategies might be required to maintain key regulators of cell migration in a functional, responsive state. Excessive ROS has also been linked to the inactivation of redox-sensitive phagosomal enzymes, such as cathepsins, dedicated to the digestion of engulfed materials (Rybicka et al., 2010). Intriguingly, macrophages lacking Nrf2 also accumulated cytosolic vacuoles, accompanied by increased cell body size (Fig. 4, E–G), which might reflect either a defect in phagosome digestion or precocious phagocytosis of corpses from the environment. Given that hypervacuolation was previously linked to defective cell migration (Evans et al., 2013), it might also contribute to the inability of *Nrf2*^*RNAi*^ macrophages to move effectively.

Another physiological task performed by macrophages in vivo is their inflammatory migration toward sites of epithelial damage. Live-imaging revealed that *Nrf2*^*RNAi*^ macrophages were desensitized to sterile epithelial wounds; unlike their wild-type counterparts, *Nrf2*^*RNAi*^ macrophages often ignored an adjacent wound (Fig. 4, H and I; Fig. S3 A; and Video 2). Although the persistence of Nrf2^RNAi^ macrophage migration was not altered (Fig. S3, B–D), Nrf2^RNAi^ macrophages migrated with a significantly reduced bias toward the wound (Fig. 4 J; and Fig. S3, B and E; directionality calculated as in Franz et al., 2018; Weavers et al., 2016b). Consequently, significantly fewer Nrf2^RNAi^ macrophages were recruited to the site of damage (Fig. 4 I and Fig. S3 F), although these macrophages moved at similar speeds to controls (Fig. S3, G and H). Our data suggest that Nrf2 activation not only sustains macrophage homeostatic migration but also boosts efficient inflammatory migration. To demonstrate that these migration defects were due to a specific requirement for Nrf2 within macrophages (rather than non-specific effects on the epithelium itself), we confirmed that *Nrf2* mRNA levels were not altered in cells other than macrophages (Fig. S3 I). Moreover, the level of *Nrf2* mRNA was not altered in embryos carrying the Nrf2^RNAi^ construct alone in the absence of a tissue-specific Gal4 driver (Fig. S3 J).

**Video 2.** **Control macrophages, but not Nrf2-RNAi macrophages, migrate towards sites of epithelial damage.** Time-lapse imaging of control (left) and stage 15 *srp > Nrf2*^*RNAi*^ macrophages (right) responding to the presence of a laser-induced epithelial wound. Wound position is marked by white asterisk. Macrophages are labeled in green (*srp* > GFP), nuclei are in red (*srp*-H2Acherry), epithelium is labeled in green (E-cadherin-GFP). A maximum projection after a 3D Gaussian blur (FIJI, radius 0.8/0.8/1) of 15 consecutive equatorial planes taken at 1 µm spacing is shown. Time stamp: minute. Related to Fig. 4.

Extracellular ROS produced by epithelial DUOX are thought to serve as important damage signals that promote immune cell recruitment to epithelial injury (Evans et al., 2015; Razzell et al., 2013; Yoo et al., 2011), although whether these ROS function as permissive cues or chemotactic attractants remains unclear. Interestingly, recent work in *Drosophila* suggests that wound-induced hydrogen peroxide diffuses into adult macrophages through Prip aquaporin-like channels (Chakrabarti and Visweswariah, 2020); Prip channels are also expressed in embryonic macrophages (Fig. S3 K), however, their role in directing macrophages to sites of injury remains unexplored. The ability of cells to sense elevated ROS in response to injury could rely on differential levels of the signal between the intracellular and extracellular compartments. Perhaps the inability of Nrf2^RNAi^ macrophages to properly detoxify intracellular ROS significantly reduces the ROS gradient between the intracellular and extracellular space and thus compromises their “orienteering” skills.

As well as clearing corpses during immune surveillance, macrophages play important roles in phagocytosing cellular debris at sites of tissue injury, and this is associated with further phagosomal ROS production (Weavers et al., 2019). Moreover, epithelial damage is also associated with elevated (Duox-dependent) ROS production at the wound margin (Razzell et al., 2013). We thus speculated that leukocytes recruited to wounds might require elevated cytoprotection and could further upregulate Nrf2. Analysis of macrophage Nrf2 (using GFP-tagged Nrf2) revealed a significant increase in macrophage Nrf2 levels following epithelial injury (Fig. 4, K and K′; and Video 3). We occasionally observed that macrophages lacking Nrf2 showed signs of DNA fragmentation following corpse uptake at wounds, indicative of apoptotic cell death (Video 4), suggesting Nrf2^RNAi^ macrophages might be more vulnerable to the build-up of ROS encountered during wound healing. These results suggest that macrophages may need to boost their Nrf2-mediated resilience above homeostatic levels to achieve protection from the elevated oxidative insult they might experience during an inflammatory response.

**Video 3.** **Nrf2 is upregulated in 18** **h pupal macrophages in response to tissue damage.** Macrophages recruited to an epithelial wound (white asterisk) increased levels of Nrf2-GFP above unwounded levels. Nrf2-GFP in green (left), macrophages are labeled in magenta (*srp*-mcherry). A maximum projection of 21 consecutive equatorial planes taken at 2 µm spacing is shown. Time stamp: minute. Scale bar: 50 µm. Related to Fig. 4.

**Video 4.** **Zoom into an epithelial wound, related to** Video 2**.** Stage 15 Nrf2^RNAi^ macrophages (lower panel) were more sensitive to the build-up of ROS experienced during clearance of debris at the wound site. Clear signs of nuclear fragmentation, suggestive of apoptotic cell death, were visible (white arrow). This behavior was not observed for macrophages of control animals (upper panel). Wound position is marked by a white asterisk. Macrophages are labeled in green (*srp* > GFP), nuclei are in red (*srp*-H2A::mcherry). A maximum projection after a 3D Gaussian blur (FIJI, radius 0.8/0.8/1) of 15 consecutive equatorial planes taken at 1 µm spacing is shown. Time stamp: minute. Related to Fig. 4.

### Leukocyte Nrf2 limits early senescence and minimizes non-autonomous collateral damage

**Figure 5.**
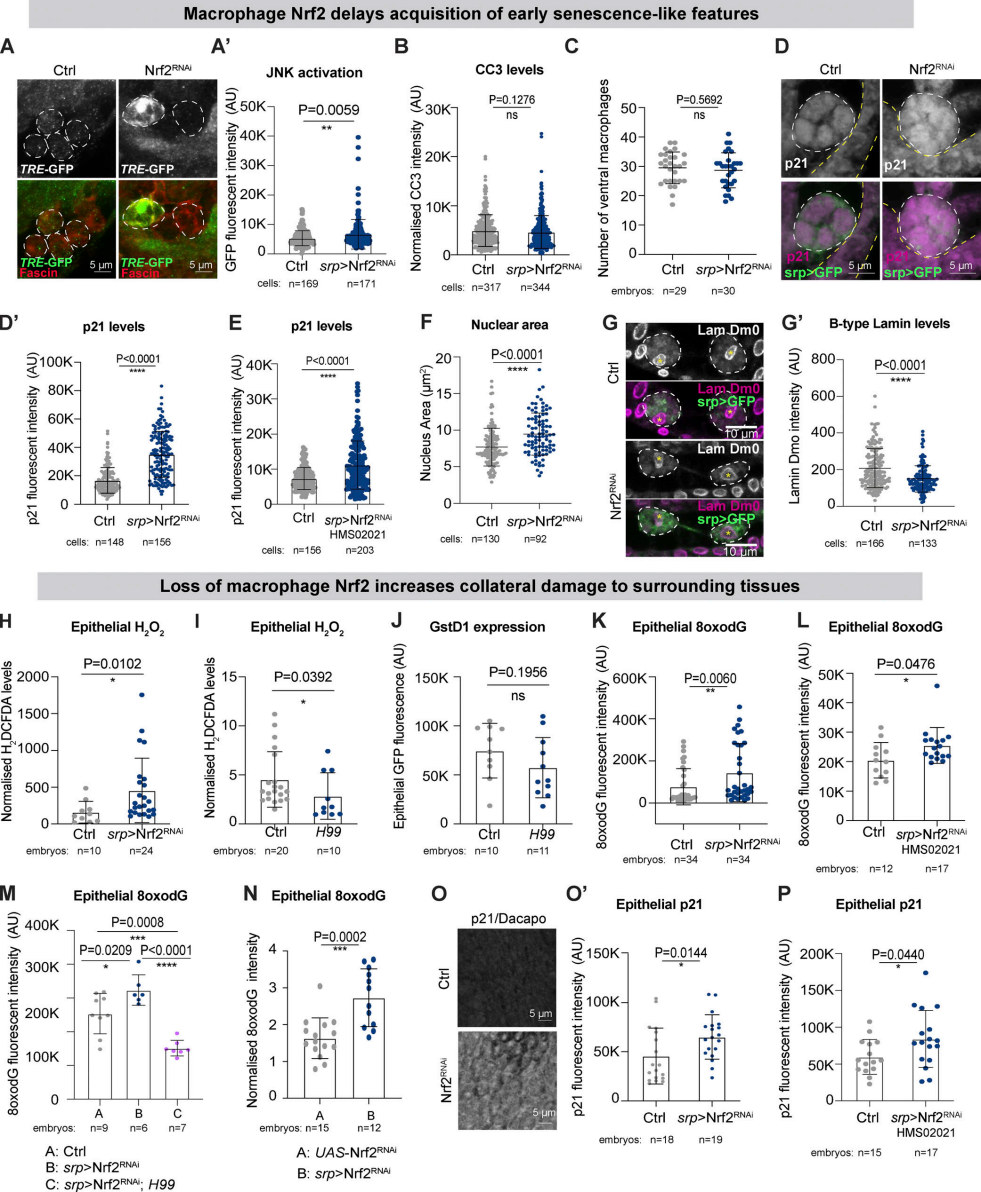
**Macrophage Nrf2 delays acquisition of early senescence-like features and limits collateral damage. (A)** stage 15 Nrf2^RNAi^ macrophages activated JNK signaling (*TRE*-GFP reporter, green) above control levels. **(B)** Apoptotic Cleaved Caspase 3 (CC3) in stage 15 control and *srp* > Nrf2^RNAi^ macrophages. **(C)** The number of ventrally localized *srp* > Nrf2^RNAi^ macrophages was indistinguishable from wild type. **(D and E)** Stage 15 *srp* > Nrf2^RNAi^ macrophages upregulated the p21/p27 homolog Dacapo (magenta). **(F and G)** Stage 15 Nrf2^RNAi^ macrophages are characterized by larger nuclei (F) and reduced levels of B-type Lamin Dm0 (G). **(H)** Levels of hydrogen peroxide (H_2_DCFDA) were increased in the epithelium upon macrophage Nrf2^RNAi^. **(I and J)** Levels of hydrogen peroxide (H_2_DCFDA) were reduced in the epithelium of *H99* embryos but activation of GstD1, *ARE*-GFP reporter was unchanged. **(K and L)** DNA oxidation (8oxodG) was significantly increased in the epithelium of *srp* > Nrf2^RNAi^ embryos. **(M)** The *H99* mutation rescued the increase in the levels of epithelial DNA oxidation (8oxodG) caused by macrophage Nrf2^RNAi^ to subcontrol levels. **(N)** DNA oxidation (8oxodG) was increased in the epithelium of *srp* > Nrf2^RNAi^ embryos when compared with embryos carrying the RNAi construct alone (*UAS*-Nrf2^RNAi^). **(O and P)** Levels of the senescence marker p21/p27 Dacapo were significantly increased in the epithelium of *srp* > Nrf2^RNAi^ embryos. Cell bodies indicated by white dashed outline. Macrophages labeled in green (*srp* > GFP, D, E) or red (fascin, A); adjacent epithelium is marked by yellow dashed lines (D and E). Data (A–D, F–H, K, and M–O) generated using Nrf2^RNAi^ Flybase ID FBtp0069370 and data (E, L, and P) generated using Nrf2^RNAi^ TRiP HMS02021. ns: not significant, *, P < 0.05; **P < 0.01, ****P < 0.0001 via Mann-Whitney test (A′, B, D′, E, F′, G, H, J, K, O′, P) or unpaired *t* test (C, I, and N), one-way ANOVA followed by Dunn’s comparison analysis (M). Images collected from 5 controls and 6 *srp* > Nrf2^RNAi^ embryos (A); 15 control, 15 srp > Nrf2^RNAi^ (B); 5 controls, 6 srp > Nrf2^RNAi^ (D); 5 controls, 7 srp > Nrf2^RNAi^ (E); 6 controls, 8 srp > Nrf2^RNAi^ embryos (F–G).

Cellular ROS have well-characterized roles regulating MAPK signaling, particularly through the c-Jun N-terminal kinase (JNK) pathway (Finkel, 2011). Since JNK signaling is highly redox-sensitive, we explored whether Nrf2-deficient macrophages exhibited altered JNK activity using a *TRE*-GFP reporter that consists of AP-1 binding sites upstream of GFP (Chatterjee and Bohmann, 2012). Nrf2^RNAi^ macrophages exhibited increased JNK activation compared with controls (Fig. 5, A and A′). Given that sustained and excessive levels of JNK signaling have been linked to apoptosis (Liu and Lin, 2005), we explored potential effects on macrophage viability. However, the level of the pro-apoptotic marker Cleaved Caspase-3 (CC3; Fig. 5 B) as well as the number of ventrally localized macrophages (Fig. 5 C) were indistinguishable from controls, arguing against apoptotic cell death.

Persistent oxidative stress and DNA damage, as well as excessive JNK activity, are well-known triggers of premature stress-induced cellular senescence (d’Adda di Fagagna, 2008; Passos and Von Zglinicki, 2006). Interestingly, Nrf2^RNAi^ macrophages showed elevated expression of the cyclin-dependent kinase (CDK) inhibitor Dacapo (Fig. 5, D and E), a *Drosophila* p21/p27 homolog previously associated with an early senescent state in flies (Ito and Igaki, 2016; Nakamura et al., 2014). p21 elevation triggers cell cycle arrest during senescence in *Drosophila* (Ito and Igaki, 2016) and is implicated in the acquisition of senescent-like phenotypes during mammalian embryonic development (Storer et al., 2013) and in murine postmitotic neurons (Jurk et al., 2012). As well as cellular hypertrophy, cells in the early stages of senescence normally exhibit increased nuclear size and a marked decrease in the levels of Lamin B1 (Lamin Dm0 in flies; Lin et al., 2022; Wang et al., 2017; Freund et al., 2012), all of which we observed in Nrf2-deficient macrophages (Fig. 4 E; and Fig. 5, F and G). Our data thus suggest that depletion of macrophage Nrf2 not only leads to the uncontrolled accumulation of key triggers of senescence (ROS, oxidative, and DNA damage) but alteration of cellular markers typically associated with early senescence.

When matured to a full senescence state, senescent cells can release secretory components such as inflammatory cytokines, matrix remodeling factors (e.g., MMP1), and growth factors in a phenomenon known as senescence-associated secretory phenotype (SASP; Ito and Igaki, 2016); in fact, JNK regulates expression of SASP factors in mammals and *Drosophila* (Ito and Igaki, 2016). However, levels of the *Drosophila*-secreted MMP, MMP1 were not elevated in stage 15 Nrf2^RNAi^ macrophages (data not shown), suggesting that Nrf2 depletion alone is insufficient for the induction of a fully senescent state associated with SASP at this stage in *Drosophila* macrophages.

Given the close proximity of phagocytes to non-immune tissues in vivo, a dysregulated immune system could compromise the health of remote organs. Indeed, recent work has shown that murine immune cells that lack the DNA repair protein Ercc1 not only undergo premature senescence but exert detrimental systemic effects on non-lymphoid tissues (Yousefzadeh et al., 2021). Senescent immune cells might release proinflammatory cytokines (and/or SASP mediators) that could contribute to the onset of a chronic inflammatory state, fueling the accumulation of collateral damage. Here, the inability of Nrf2-deficient macrophages to properly detoxify ROS could have marked non-autonomous effects on surrounding tissues, perhaps through the release of ROS (e.g., hydrogen peroxide) that diffuse readily across membranes through aquaporin-like channels (Bienert et al., 2006; Chakrabarti and Visweswariah, 2020). Indeed, we found that loss of macrophage Nrf2 was associated with significant, non-autonomous accumulation of hydrogen peroxide in the adjacent epithelium (Fig. 5 H; also see Fig. S1 G). Interestingly, epithelial ROS levels (but not Nrf2 activity) were reduced in *H99* mutant embryos compared with controls (Fig. 5, I and J), suggesting that even wild-type macrophages contribute to the non-autonomous epithelial accumulation of ROS downstream of apoptotic corpse engulfment.

Loss of macrophage Nrf2 was also associated with elevated non-autonomous bystander damage to surrounding tissues, as indicated by the increased level of epithelial DNA oxidation (Fig. 5, K and L; also see Fig. 2 D). Strikingly, abrogation of apoptosis via *H99* not only completely rescued this non-autonomous epithelial damage in animals with Nrf2-deficient macrophages (Fig. 5 M; also see Fig. 2 F) but significantly reduced the levels of bystander damage below that of control animals (Fig. 5 M; also see Fig. 2 F). These data suggest that epithelial oxidative damage occurs downstream of macrophage-mediated corpse clearance, but that this is normally restrained by macrophage Nrf2. Moreover, levels of epithelial damage were significantly increased in *srp* > Nrf2^RNAi^ animals compared with those carrying the Nrf2^RNAi^ construct alone, excluding leaky non-specific expression of the RNAi construct (Fig. 5 N). The epithelium of embryos lacking macrophage Nrf2 also exhibited elevated levels of the *Drosophila* p21/p27 homolog (Dacapo; Fig. 5, O and P), suggesting that persistent ROS-induced epithelial damage might trigger the acquisition of cellular features typically associated with early senescence, despite non-immune tissues retaining normal levels of Nrf2 (Fig. S3 I).

Given the profound cell-autonomous and systemic alterations caused by the loss of macrophage Nrf2, the ability to boost leukocyte cytoprotection could have enormous clinical benefits. Since wild-type macrophages even elevate ROS levels non-autonomously in adjacent tissues during immune surveillance, perhaps further activation of Nrf2-mediated ROS detoxification in the innate immune system could benefit host fitness. Therapeutic augmentation of phagocyte cytoprotective activity could particularly help individuals suffering from inflammatory conditions associated with elevated ROS production or those patients with a prematurely aged immune system. Nevertheless, given that recent work suggests constitutive elevation of cytoprotection in many cell types can have detrimental effects (Hiebert et al., 2018; Kucinski et al., 2017), any therapeutic augmentation of cytoprotection will need to be carefully controlled. Indeed, there is increasing evidence that basal levels of cellular stress even have beneficial hormetic effects by triggering adaptive responses that promote stress resistance (Fischer and Ristow, 2020).

Our work highlights the urgent need to define the complex networks of immune “resilience” pathways and gain an in-depth mechanistic understanding of their systemic impact on organismal aging. Phagocytes generate significant ROS throughout development, homeostasis, and inflammation, but to resist the costly consequences on cell health, immune cells simultaneously activate powerful protective machinery to mitigate the damage. However, protection from indiscriminate ROS is likely only one arm of this complex immune cytoprotective network; indeed, the recent targeted deletion of the DNA repair protein Ercc1 in hematopoietic cells accelerated immune and systemic aging (Yousefzadeh et al., 2021). The unrivaled potential for in vivo large-scale screening in *Drosophila* could allow the design of novel therapies to finely tune (or even reactivate) immune cytoprotection to mitigate deleterious effects of an aging immune system and tackle age-related multimorbidities.

## Materials and methods

### *Drosophila* stocks and genetics

*Drosophila* stocks were raised and maintained on Iberian food according to standard protocols. For crosses, adults were placed in cages and kept in an incubator at 25°C and controlled humidity, unless otherwise stated. Embryos were collected on standard apple juice plates prepared in-house. Embryos of the appropriate genotype were selected based on the presence of fluorescent proteins or the absence of fluorescent balancer chromosomes carrying fluorescent markers. The following fly stocks were used: w1118 (#5905; Bloomington), srp-Gal4 (Brückner et al., 2004), crq-Gal4, UAS-GFP, GstD, *ARE*-GFP (Sykiotis and Bohmann, 2008), *4XARE*-GFP-16 (Nrf2 activity reporter [Chatterjee and Bohmann, 2012]), *srp* > mcherry::Moesin (Moreira et al., 2010), Df(3)H99 (#1576; Bloomington [White et al., 1994]), UAS > nuclear Red Stinger (Barolo et al., 2004), UAS-Nrf2^RNAi^ (Chatterjee and Bohmann, 2012; gift from Ioannis Trougakos, University of Athens, Greece; Flybase ID FBtp0069370), UAS-Nrf2^RNAi^ TRiP HMS02021 (#40854; Bloomington), UAS-Luciferase^RNAi^ TRiP JF01355 (#31603; Bloomington), UAS-LexA^RNAi^ TRiP HMS05772 (#67947; Bloomington), UAS-NOX^RNAi^ (VDRC v102559), UAS-NOX^RNAi^ TRiP HMS00691 (#32902; Bloomington), Nrf2-GFP (#38631; Bloomington), E-cadherin GFP (#60584; Bloomington [Oda et al., 1998]), *srp*-H2Acherry and *srp*-mcherry (Gyoergy et al., 2018),UAS-Duox^RNAi^ (Ha et al., 2005) UAS-GPH (Pickering et al., 2013), UAS-Dp110^D954A^ (Leevers et al., 1996), UAS-PI3K^RNAi^ TRiP JF02770 (#27690; Bloomington), UAS-Ryr^RNAi^ (TRiP JF03381 [#29445; Bloomington] and TRiP HM05130 [#28919; Bloomington]), UAS-GcaMP3 (gift from John Gillespie, University of Bristol, UK), and *TRE*-GFP (Chatterjee and Bohmann, 2012). *Drosophila* mutant and transgenic lines were obtained from Bloomington Stock Centre (NIH P40OD018537) or the Vienna *Drosophila* Resource Centre (VDRC, www.vdrc.at), unless otherwise stated.

### Dissection and imaging of *Drosophila* pupae

Pupae were staged (18 h APF) in regular vials at 25°C. Animals were carefully dissected out of their pupal case using forceps as described before (Weavers et al., 2018) and mounted on glass-bottomed dishes (Cat. #P35G-0-10-C; MatTek) for live imaging. For wounding experiments, wounds were generated on pupal wings by using a Nitrogen-pumped laser point ablation laser tuned at 435 nm (Andor Technologies). Samples were imaged on a TSC SP8 confocal microscope using a 40/1.3 oil immersion objective.

### Live imaging of embryos and wounding

Embryos of an overnight collection were dechorionated in 50% bleach for 1 min and extensively washed in water. Embryos at stage 15 of embryonic development (600–800 min AEL [Vässin et al., 1985]) were mounted ventral-side up on double-sided scotch tape on a glass slide in 10S Voltalef Oil (Cat. #24627.188; VWR). For wounding experiments, wounds were generated by using a Nitrogen-pumped Laser point ablation laser tuned at 435 nm (Andor Technologies). Samples were imaged on a TSC SP8 confocal microscope using a 63/1.4 oil immersion objective. Imaging processing was performed using ImageJ (NIH) and Adobe Illustrator software.

### Injection of fluorescent dyes and drug treatment

Embryos of an overnight collection were dechorionated in 50% bleach for 1 min and extensively washed in water. Stage 15 embryos were mounted on double-sided scotch tape on a glass slide and dehydrated for a minimum of 10 min before being covered in Voltalef Oil. Embryos were microinjected with the desired fluorescent probe for the detection of ROS. A 30 μM stock of dihydroethidium (Cat. #D11347; DHE, Invitrogen, Molecular Probes) or a 10 μM stock of 2',7′-dichlorodihydrofluorescein diacetate (Cat. #D399; H2DCFDA, Invitrogen, Molecular Probes,) was prepared in DMSO (Cat. #D2438; Sigma-Aldrich) and diluted 1:50 in 1× PBS for injection. Uninjected embryos were imaged as a negative control to normalize for background fluorescence. For detection of ROS at the early stage 12 of embryonic development, embryos were incubated in a 1:1 solution DHE/Heptane and incubated for 30 min at room temperature under constant agitation. Following incubation, embryos were mounted for live imaging as described above. 1 mM stock solutions of Thapsigargin (Cat. #1138; Tocris) and Ryanodine (Cat. #1329; Tocris) were dissolved in DMSO. Stocks were diluted to 1 and 200 μM, respectively in Ringer’s solution (128 mM NaCl, 2 mM KCl, 35.5 mM sucrose, 5 mM HEPES, and 4 mM MgCl_2_). DMSO (Sigma-Aldrich) was added to Ringer’s solution and used as a negative control to normalize for background fluorescence.

### Immunostaining

For immunofluorescence experiments, embryos of an overnight collection were fixed for 20 min in a 1:1 solution of heptane:4% formaldehyde (in 1× PBS), on a rocking platform. Following fixation, formaldehyde was substituted with methanol. Samples were shaken vigorously to help devitillinization before being stored in fresh methanol at −20°C or directly processed for immunofluorescent staining. Samples were rehydrated by washing three times in 0.1% Tween-20 in 1X PBS (PBT), 15 min each, and then incubated for 1 h at room temperature in a blocking solution of 5% donkey serum (DS) prepared in PBT. Embryos were incubated overnight at 4°C in primary antibodies, in blocking solution, used at the following dilutions: mouse, anti-fascin antibody 1:50 (clone sn 7c, DSHB), rabbit, anti-γH2AvD (1:500; GeneTex), mouse, anti-8oxodG (1:200; Trevigen), goat, anti-GFP (1:200; ab 6673; Abcam), rabbit, anti-GFP (1:100; A11122; Invitrogen,), mouse, anti-p21/Dacapo (1:100, NP1, DSHB), rabbit, anti-Cleaved Caspase-3 (1:50; #9661; Cell Signaling), mouse, and anti-Lamin Dmo (1:100; ADL67, DSHB). The following morning, the staining solution was removed and the samples were washed three times in PBT for a total of 45 min. Samples were then incubated for 2 h in secondary antibodies at room temperature. All secondary antibodies were purchased from Jackson Laboratory and used 1:200 in the blocking solution. If necessary, biotinylated secondary antibodies (1:200; Vector laboratories) and streptavidin conjugated with fluorophores (1:200; Vector laboratories) were used to amplify the signal. Samples were mounted in Mowiol 4–88 mounting medium (Cat. #8138; Sigma-Aldrich) supplemented with DABCO anti-fading agent (Cat. #D27802; Sigma-Aldrich). Samples were imaged on a TSC SP8 confocal microscope using 40/1.3 and 63/1.4 oil immersion objectives. Imaging processing was performed using ImageJ (NIH) and Adobe Illustrator software.

### Flow cytometry

Embryos were collected overnight at 25°C from the desired fly lines. *w*^*1118*^ embryos served as a negative control. Dissociation of embryos was performed using a protocol adapted from Gyoergy et al. (2018). Briefly, embryos were dechorionated in 50% bleach for 1 min and extensively washed in water. For each genotype, a minimum of 100 embryos at stage 14–15 of embryonic development were selected under a dissecting scope and gently transferred into a dounce homogenizer containing 500 μl of freshly prepared, ice-cold Seecof buffer (6 mM Na2HPO_4_, 3.67 mM KH2PO_4_, 106 mM NaCl, 26.8 mM KCl, 6.4 mM MgCl_2_, and 2.25 mM CaCl_2_ at a pH of 6.8). All the subsequent steps were performed on ice or at 4°C. Embryos were homogenized in Seecof buffer with seven gentle vertical strokes of a loose pestle. The resulting cell suspension was collected in a fresh tube and spun down at 500 rpm for 5 min to remove debris. Subsequently, the supernatant was spun down at 1,250 rpm for 10 min. After this centrifugation step, the pellet was resuspended in 500 μl Schneider’s medium (S0146; Sigma-Aldrich) supplemented with 8% fetal bovine serum (FBS, F7524; Sigma-Aldrich) and filtered through a 40-μm nylon mesh. Cell sorting was performed on BD FACS Aria II (Becton Dickinson) fitted with a 100 μm nozzle. Cells were gated according to forward and side scatters, and dead cells were excluded based on the staining with the LIVE/DEAD permeable dye Draq7 (ab109202; Abcam). GFP and mCherry signals were detected through 530-40 and 610-20 filters, respectively. Cells were sorted in 1.5 ml Eppendorf tubes and immediately resuspended in the appropriate buffer for subsequent use. FCS files generated from sorting were analyzed using FlowJo_v10.6.2 software (Becton, Dickinson & Company).

### RNA extraction and RT-qPCR

RNA was extracted from sorted cells using the RNeasy Plus Micro Kit (74034; Qiagen). Briefly, cells were resuspended in 350 μl of Lysis buffer and the suspension was passed through a 30 G needle to facilitate lysis. Recovery of RNA was performed following the manufacturer’s instructions. RNA was eluted in 100 μl of RNase-free warm water. RNA was precipitated by adding 0.10 vols of 3 M sodium acetate and 2 vol of 100% of ice-cold ethanol to each sample and incubating overnight at −80°C. The day after, 1 μl of 20 mg/ml glycogen (R0551; Thermo Fisher Scientific) was added to each sample. Samples were centrifuged at 13,000 rpm for 30 min at 4°C. RNA pellet was washed twice with 500 μl of ice-cold 75% ethanol and airdried. RNA was resuspended in 20 μl of RNase-free water. RNA quality and concentration were assessed on Agilent ScreenTape System.

For real-time (RT) qPCR analysis, an equal amount of RNA was reverse transcribed using the Maxima First Strand Synthesis kit (K1671; Thermo Fisher Scientific) following the manufacturer’s instructions. Relative quantification of gene expression was carried out on a QuantStudio 3 Real-Time PCR System using PowerUp Syber Green master mix (A25741; Thermo Fisher Scientific). Data analysis was performed using the 2^−(ΔΔCt) values method using multiple housekeeping genes as previously described (Hellemans et al., 2007; Vandesompele et al., 2002). Primers used for Real-Time qPCR were purchased from Eurofins and their sequence is listed below: *α-tubulin 84B*, 5′-CAC​ACC​ACC​CTG​GAG​CAT​TC-3′ and 5′-CCA​ATC​AGA​CGG​TTC​AGG​TTG-3′; *rpl32*, 5′-AGC​ATA​CAG​GCC​CAA​GAT​CG-3′ and 5′-TGT​TGT​CGA​TAC​CCT​TGG​GC-3′; *GFP*, 5′-AAG​CTG​ACC​CTG​AAG​TTC​ATC​TGC-3′ and 5′-CTT​GTA​GTT​GCC​GTC​GTC​CTT​GAA-3′; *Nox*, 5′-CAT​CGC​GGT​TCA​GTG​TCG​T-3′ and 5′-ACT​GCT​GGT​TGA​TGG​GTT​GC-3′; *Duox*, 5′-TCC​TAT​TCG​GAT​GGG​GTT​TAC​G-3′ and 5′-CAG​TCC​GGT​TGA​ACT​TTG​ACC-3′; *nrf2*, 5′-CTG​CAT​CGT​CAT​GTC​TTC​CAG​T-3′ and 5′-AGC​AAG​TAG​ACG​GAG​CCA​T-3′; *gstD1*, 5′-CCG​TGG​GCG​TCG​AGC​TGA​ACA-3′ and 5′-GCG​CGA​ATC​CGT​TGT​CCA​CCA-3′ (Rahman et al., 2013); *gclM*, 5′-CGG​TGC​AAA​GTG​TAT​TTA​AGT​GG-3′ and 5′-TGG​TCG​GTA​TCA​TGG​TGA​CA-3′ (Cheng et al., 2021); prip, 5′-CTG​GAA​TGT​TGG​TCG​CAG​GA-3′ and 5′-GCA​GGA​GTC​CCA​GGA​AGA​AC-3′.

### Data analysis

All images and movies were processed and analyzed using ImageJ (NIH). For quantification and comparison of fluorescent intensity of immunostaining and DHE injection, images were opened in ImageJ and converted into 16-bit images. For each Z-stack, sum-intensity projections were created and a line was drawn around each cell body manually, using the free-hand tool. For each region of interest, integrated pixel intensity was measured using the Analyse/Measure tool in the channel of interest. Pixel intensities were normalized to background values taken from cell-free regions. Data were normalized to negative controls (uninjected embryos, non-transgenic wild-type *w*^*1118*^ embryos, or staining without secondary antibody) and values were plotted on Prism as fold-change over the negative control. To quantify the accumulation of the PIP3 (GPH) reporter on the membrane of newly formed phagosomes, sum-projections of macrophages from live-imaging movies were made from Z-stacks using ImageJ. The free-hand tool was used to draw a line around the newly formed phagosome and the Analyse/Measure function tool was used to quantify pixel intensity along the line. The width of the line was set at 2. Background fluorescence was calculated by measuring pixel intensity from an area of the lamellipodium where no phagosomes were present. Data on the graphs are therefore shown as fold-change over background levels. Expression of cytosolic GFP (cytoGFP) was used as a control. For analysis of macrophage motility, cells were tracked using the automated cell tracking protocol of Imaris or using manual tracking on ImageJ. To quantify the number of macrophages responding to wounds at a specific time point, the total number of cells in contact with the wound margin or within the wound area was calculated from z-stack projections.

All statistical comparisons and graphical representations were generated using Prism8 for Mac (Graphpad). Data in the graphs are represented as mean ± SD, unless otherwise stated. Statistical analysis was performed as specified in each figure legend and the appropriate statistical test was selected after testing samples for normality. P values less than 0.05 were considered significant. *n* numbers and exact P values are reported on each graph.

### Online supplemental material

Fig. S1 shows the activation of Nrf2 protects embryonic macrophages from excessive ROS accumulation. Related to Fig. 1. Fig. S2: A PI3K, Calcium, and NOX axis drive macrophage Nrf2 activation. Related to Fig. 3. Fig. S3: Macrophage Nrf2 is required for the timely detection of epithelial wounds in vivo. Related to Fig. 4. Video 1: PIP3 accumulates on the membrane of a nascent phagosome. Related to Fig. 3. Video 2: Control macrophages, but not Nrf2-RNAi macrophages, migrate toward sites of epithelial damage. Related to Fig. 4. Video 3: Nrf2 is upregulated in 18 h pupal macrophages in response to tissue damage. Related to Fig. 4. Video 4: Zoom into an epithelial wound, related to Video 2.

## Data Availability

The data underlying Figs. 1, 2, 3, 4, and 5 are available from the corresponding author upon reasonable request.
